# Pandemic *Vibrio parahaemolyticus* O3:K6, Europe

**DOI:** 10.3201/eid1108.050322

**Published:** 2005-08

**Authors:** Jaime Martinez-Urtaza, Lourdes Simental, David Velasco, Angelo DePaola, Masanori Ishibashi, Yoshitsugu Nakaguchi, Mitsuaki Nishibuchi, Dolores Carrera-Flores, Carmen Rey-Alvarez, Anxela Pousa

**Affiliations:** * Universidad de Santiago de Compostela, Santiago de Compostela, Spain;; † Complexo Hospitalario Universitario Juan Canalejo, A Coruña, Spain;; ‡ US Food and Drug Administration, Dauphin Island, Alabama, USA;; § Osaka Prefectural Institute of Public Health, Osaka, Japan;; ¶ Kyoto University, Kyoto, Japan;; # Consellería de Sanidade, Santiago de Compostela, Spain

**Keywords:** O3:K6, Pandemic clone, PFGE, AP-PCR, food safety, foodborne outbreak

**To the Editor:** *Vibrio parahaemolyticus* is a halophilic member of the genus *Vibrio* that inhabits temperate and tropical marine environments worldwide. Strains that produce the thermostable direct hemolysin or the thermostable direct hemolysin-related hemolysin, which are encoded by *tdh* and *trh* genes, respectively, are considered pathogenic. While almost all clinical strains have these virulence factors, these strains represent <1% of all environmental strains.

Recently, *V*. *parahaemolyticus* infections have increased globally; they are usually associated with eating raw or undercooked seafood. *V*. *parahaemolyticus* is the leading cause of seafood-associated bacterial gastroenteritis in the United States (1) and causes approximately half of the foodborne outbreaks in some Asian countries (2). In 2001, the Scientific Committee on Veterinary Measures Relating to Public Health of the European Commission concluded that *V*. *parahaemolyticus* outbreaks are rarely reported in Europe (3). Because the risk of *V*. *parahaemolyticus* infection is extremely low in Europe, the organism has been excluded from the European Network for Epidemiologic Surveillance and Control of Communicable Diseases and from Microbiologic Surveillance System for Infectious Gastroenteritis. *V*. *parahaemolyticus* is also excluded from the European applicable microbiologic requirements for shellfish-harvesting areas and ready-to-eat seafood.

However, data obtained after an exhaustive review of clinical journals published in Spain and from unreported cases of *V*. *parahaemolyticus* infections identified at Spanish hospitals have shown that *V*. *parahaemolyticus* infections in Spain are more common than previously assumed. This organism was isolated from patients with gastroenteritis in Barcelona (1986, 1987, and 1999), Zaragoza (1993), and Madrid (1998 and 2000). In Galicia (northwestern Spain) alone, where most Spanish shellfish are produced, 84 cases of *V*. *parahaemolyticus* infection were identified retrospectively from hospital records from 1997 to 2000. A single outbreak of 64 cases in 1999 was associated with oyster consumption (4). Most Spanish clinical isolates were serotype O4:K11, and pulsed-field gel electrophoresis (PFGE) analysis demonstrated these to be a unique clone distinct from Asian and American clinical strains (5).

In July 2004, a *V*. *parahaemolyticus* outbreak of 80 illnesses occurred in A Coruña, Spain. All the case-patients attended weddings in the same restaurant. *V*. *parahaemolyticus* was isolated from stool samples of 3 patients. The outbreak isolates were characterized by serotyping, polymerase chain reaction (PCR) for species-specific genes (Vp-*toxR* and *tlh*), virulence-related genes (*tdh* and *trh*), and group specific (GS)-PCR (a PCR method to detect the pandemic clone). Two isolates belonged to the serotype O3:K6, while the remaining isolate was O3:K untypeable. All 3 isolates had the *toxR*, *tlh*, and *tdh* genes, lacked the *trh* gene, and were positive for the GS-PCR assay to detect pandemic strains. These results unequivocally linked the outbreak isolates to the O3:K6 pandemic clone of *V*. *parahaemolyticus*. To confirm the relationship with the pandemic clone, the outbreak isolates were additionally subjected to DNA fingerprinting analyses. PFGE and arbitrarily primed PCR analyses showed that these isolates exhibited a pattern indistinguishable from those of pandemic strains from Asia. The epidemiologic investigation associated with the outbreak identified the boiled crab eaten in the restaurant as the most probable source of the infection. Live crabs were imported to Spain from the United Kingdom, processed under unhealthy conditions, and stored at room temperature for several hours before they were eaten. All the seafood eaten at the weddings was harvested in Europe, and no imported food was eaten or handled in the restaurant.

Pandemic O3:K6 clone of *V. parahaemolyticus* appeared in Asia around 1996 (6). Since its emergence, it has accounted for most *V*. *parahaemolyticus* infections in Asia. It spread to the United States in 1998 (7) and more recently to Chile (8), where it has caused hundreds of infections, resulting in the first *V*. *parahaemolyticus* pandemic in history (9). We report the first evidence that it has been introduced to Europe. The emergence of this virulent serotype in Europe is a public health concern and emphasizes the need to include *V*. *parahaemolyticus* in microbiologic surveillance and reexamine control programs for shellfish-harvesting areas and ready-to-eat seafood.
